# Socio-economic position and changes in 24-h movement behaviors during the retirement transition

**DOI:** 10.1186/s44167-025-00087-7

**Published:** 2025-10-16

**Authors:** Nina Vansweevelt, Jannique van Uffelen, Filip Boen, Kristin Suorsa, Jan Seghers

**Affiliations:** 1 Department of Movement Sciences, KU Leuven, Tervuursevest 101 bus 1500, 3001 Leuven, Belgium; 2 Department of Public Health, Turku University Hospital, University of Turku, Turku, Finland; 3 Centre for Population Health Research, University of Turku and Turku University Hospital, Turku, Finland

**Keywords:** Socio-economic status, Physical activity, Sedentary behavior, Sleep, Compositional data analysis, 24-h movement behaviors, Retirement, Ageing

## Abstract

**Background:**

The retirement transition provides a window of opportunity for the optimization of activity, sedentary and sleep behaviors. Identifying groups at risk for non-favorable changes is important in this matter. There are indications that lower socio-economic position (SEP) adults might be more prone to non-favorable changes. However, previous studies mainly used self-reported behaviors and only one indicator of SEP. The purpose of the present study was therefore to examine the association of SEP with changes in device-measured physical activity, SB and sleep during the retirement transition in adults in Flanders (Belgium) in a longitudinal study.

**Methods:**

The behaviors were measured pre-retirement and at three, six and twelve months post-retirement with a wrist-worn accelerometer (*n* = 96). The raw acceleration data were processed using the R package GGIR and analysed using compositional data analysis in linear mixed models including the SEP indicators education, occupation and income. Additionally, changes in intensity gradient and average acceleration were examined.

**Results:**

The results showed that on average, physical activity was stable, sleep increased (+ 18 min) and SB decreased (− 15 min). The intensity gradient and average acceleration did not change significantly. The higher income group had significantly more favorable changes in movement behaviors compared to the lower income group. More specifically, they increased physical activity and shifted towards more intense physical activity, while the lower income group did not. The higher education and occupation groups showed a non-significant trend towards more favorable changes. The changes occurred mainly between pre-retirement and three months post-retirement and were relatively stable afterwards.

**Conclusion:**

The behaviors shifted towards more healthy behaviors in general, with stable physical activity, a decrease in SB and an increase in sleep. The changes in the behaviors were more favorable for retirees with higher SEP compared to retirees with lower lower socio-economic position. There seems to be a need for strategies to improve 24-h movement behaviors of lower SEP adults during the retirement transition. However, our results are based on a small sample and should be validated in larger studies.

**Supplementary Information:**

The online version contains supplementary material available at 10.1186/s44167-025-00087-7.

## Background

The transition from work to retirement is likely to impact a person’s daily physical activity (PA) levels, sedentary behavior (SB), and sleep duration as the absence of work hours provides an opportunity to redistribute these behaviors in a 24-h day [1]. Literature reviews, mainly based on self-reported behaviors, have found that total PA and SB decrease during the retirement transition [2, 3], while sleep duration increases [4]. These findings have been confirmed in device-based observational studies, predominantly conducted in the Nordic countries, which have reported similarly a decrease in total PA [5–7], a decrease in SB [5] and an increase in sleep duration during the retirement transition [8, 9].

The retirement transition provides a window of opportunity for health promotion initiatives, since daily life and daily routines change naturally and new habits could potentially be incorporated in the new, retired life. Therefore, it is essential to examine factors associated with the patterns of change. This will allow for identification of at-risk subgroups with less favorable changes who will benefit the most from health promotion initiatives to optimise their health as they age. A key socio-demographic factor influencing retirement-related changes in PA and SB might be an individual’s socio-economic position (SEP) [10]. More specifically, adults with higher SEP seem to have more favorable changes in PA and SB when they retire compared with adults with lower SEP. In addition, several long-term cohort studies have found that differences in leisure-time PA between occupational class groups and educational groups widen during the ageing process from midlife to older age [11–14]. Furthermore, a systematic review of 45 observational studies concluded that SEP, determined by educational level or income/wealth, is associated with healthy aging, when the latter is operationalized as a multidimensional construct [15].

SEP is a complex multifaceted concept covering several aspects of social stratification [16]. It is mostly measured by indicators such as education, occupation and/or income or a combination thereof [16]. Although it is recommended to use multiple SEP indicators in studies, the current evidence about the role of SEP in the changes in movement behaviors during the retirement transition is limited by the use of only one SEP indicator, in the device-based studies even always occupation, thereby reducing the complex multifaceted concept of SEP to one dimension.

A further limitation of previous studies is that many have investigated the changes in PA, SB and/or sleep separately, without taking into account the interdependence of the behaviors. After all, every moment of a 24-h day is allocated to either sleep, PA or SB. This means that, for example, when sleep duration increases during retirement, there has to be automatically a decrease in PA or SB, because there are only 24 h in a day. However, including all three behaviors in traditional multivariate analyses has not been feasible because these 24-h time budget data exhibit perfect multicollinearity. To overcome this problem, applying compositional data analysis (CoDA) [17] is a solution that treats the data as compositions, after which multivariate analysis can be used without the problem of multicollinearity.

Further, almost all previous research that used device-based movement behavior measurements to investigate changes during retirement has been conducted in Nordic countries, more specifically in Finland and Sweden, and the findings might not be generalizable to other countries or regions due to different cultural and environmental factors [18]. Research on this topic outside of the Nordic countries was based on self-reported measures of (one of) the behaviors, which are prone to recall bias and often focused only on specific domains within the behaviors (e.g., television viewing instead of total SB and leisure time PA instead of total PA). Device-based measurements of PA, SB and sleep usually differ substantially from self-reported measures, which might be due to the device-based measurements including more incidental PA that is performed less consciously [19, 20]. This discrepancy between device-based and self-report measurements might be important during the transition to retirement since daily activities change substantially, leading to a potential change in incidental PA.

The aim of the present study was to address the abovementioned limitations in the existing literature by examining the association of multiple SEP indicators with the changes in device-based PA, SB and sleep during the retirement transition in adults in Flanders (Belgium), using compositional data analysis (CoDA). Based on the literature, we hypothesize that, (1) regardless of SEP, PA and SB will decrease while sleep will increase during the retirement transition and (2) that PA changes will be more favorable for higher SEP adults.

## Methods

The STROBE cohort reporting guidelines were used (Supplementary file 1) [21].

### Study design

The present study adopted a longitudinal observational design with four measurement points: before retirement (one to six months prior to retirement) and after retirement (three, six and twelve months).

### Study population

A convenience sample was recruited in Flanders (Belgium), including Dutch-speaking participants who self-reported intention to retire between January 2022 and March 2023. Retirement was defined as a substantial decrease of working hours at the end of the working career. Recruitment took place through social media, employers, social health assurances and by word of mouth. Exclusion criteria were (1) retiring because of health reasons or after long-term incapacitation for work and (2) retirement of professional athletes.

This study was approved by the Social and Societal Ethics Committee of KU Leuven (G-2021-3176) and all participants provided written informed consent.

### Sociodemographic characteristics

The highest educational attainment was classified based on the International Standard Classification of Education 2011 (ISCED) [22]. This was categorised into ISCED class 0–4, including low and intermediate education (‘lower education’) and ISCED class 5–8, including participants with tertiary education degree (‘higher education’). The occupations were classified based on the International Standard Classification of Occupations 2008 (ISCO-08) [23] and categorised into two groups: classes with lower skill level (‘manual classes’) (ISCO classes 5–9, e.g. practical nurses, shop keepers, cashiers, cooks, maintenance workers, etc.) and classes with higher skill level (‘non-manual classes’) (ISCO classes 1–4, e.g. managers, teachers, engineers, doctors, nursing professionals, technicians, administrative workers, etc.). The participants’ household disposable income range was surveyed at the last measurement, i.e., 12 months after retirement. This household disposable income range was post hoc equivalised for household size based on the OECD-modified scale [24]. The range borders were divided by 1.5 for participants living together with one other adult person. Afterwards, the ranges of incomes were allocated to mainly below (‘lower income’) or above (‘higher income’) the median equivalised disposable income for Flanders (Belgium), the region where the study participants were recruited, in 2023 (€31 102 yearly) (EU-SILC 2023 Flanders (Belgium), Statbel), the year when this question was asked to the majority of the participants (details in Supplementary File 2).

### Accelerometer measurements

For the first measurement, a researcher of the team met in person with each participant at the university, at the participant’s home or at the participants workplace. For the follow-up measurements, participants could receive the accelerometer via mail as well, with a prepaid envelope to return the accelerometer to the university.

Triaxial Actigraph wGT3X-BT accelerometers were initialised using the accompanying software Actilife (version 6.13.4)(ActiGraph, Pensacola, Florida, US). The sample rate was set to 100 Hz, all LED lights and the ‘idle sleep mode’ were disabled. The participant was instructed to wear the device on the non-dominant wrist for seven consecutive 24-h cycles, except for bathing, showering, swimming or other water activities. The wrist wear location was chosen because this has been associated with the highest wear time [25] and allows to analyse all 24-h behaviors, including sleep. A paper log diary was given to the participant to complete every day of the measurement week at what time they woke up and at what time they turned the lights out to go to sleep.

The raw data were analysed with the R package GGIR, version 3.0.5, which includes validated methods to analyze multiday accelerometer data and provides estimates of PA, SB and sleep parameters [26–29]. A detailed description of the setttings in GGIR can be found in Supplementary file 3.

Using established cut-points, SB was defined as acceleration lower than 44.8 m*g*, light PA (LPA) was defined as acceleration between 44.8 m*g* and 100.6 m*g* and moderate-to-vigorous PA (MVPA) was defined as every epoch with an acceleration value higher than 100.6 m*g* [30, 31].

Additionally, to complement the data on time spent in each 24-h movement behavior based on cut-points between intensities, the intensity gradient and average acceleration were calculated according to the method by Rowlands et al. (2018) [32]. The intensity gradient is the magnitude of the negative slope of the relationship between the natural log of intensity of behavior vs. time spent in that intensity. A lower (more negative) value means that less time is accumulated in midrange and higher intensities. The average acceleration is simply the average value of the ENMO (expressed in m*g*) over each 24-h window. These outcomes provide a protocol- and population-independent indication of the intensity distribution continuum of 24-h behaviors of the participants as well as the total volume of participants’ activity.

### Statistical analysis

The proportion of time spent in each behavior was treated as compositional data. The analyses were conducted using the statistical software Rstudio (version 2024.04.2), more specifically the packages ‘lme4’ [33], ‘lmerTest’ [34], ‘compositions’ [35], ‘robCompositions’ [36], ‘emmeans’ [37], ‘multcomp’ [38] and ggplot2 [39].

The compositional means of minutes spent in the behaviors were calculated as the geometric means of the data and were rescaled to sum up to 1440 min (24 h). The compositional differences between pre-retirement and twelve months after retirement were calculated for each participant via perturbation [40]. This is a compositional operation similar to addition or subtraction but in a vector space structure. After this, the percentages were rescaled to 24 h and expressed in minutes for convenient interpretation.

For the compositional data analysis (CoDA), as a first step, isometric log-ratios were calculated to convert the compositional data into the real space, which allows standard statistical methods to be used [41]. More specifically, the present study used balance coordinates as type of isometric log ratios. The movement behaviors assigned to each coordinate were decided based on sequential binary partition [42, 43]. The first coordinate exists of active behaviors (LPA + MVPA) versus passive behaviors (SB + sleep). The second coordinate entails LPA versus MVPA, while the third coordinate is defined as SB versus sleep. These coordinates were calculated for each participant at each measurement time.

As a next step, four separate linear mixed models with full maximum likelihood estimation were fitted for each of these three coordinates to investigate the change of the 24-h behaviors over time [44]. The first model for each coordinate included only time (pre-retirement and three, six and twelve months after retirement) as predictor at level one. Hence, this model investigates the main effect of time on the coordinate. The second model added education as a predictor and the time x education cross-level interaction. The third model included occupation instead of education as a predictor and the time x occupation interaction. Similarly, the fourth model included income as a predictor and the time x income interaction.

In addition to the models that investigated the effect of time and SEP on the three CoDA coordinates, similar models were fitted to investigate the effect of time and SEP on the intensity gradient and average acceleration. All models included random intercepts, taking into account that each participant had a different baseline behavior composition.

The normality of the residuals was visually inspected on Q-Q plots and histograms of the residuals. Extreme outliers of the residuals were checked and removed in case of non-normality. Afterwards, the results with and without outliers were compared. If outliers were not present, or their removal did not normalize the residuals, the variable in question was transformed. In that case, analyses were conducted with both the non-transformed and transformed variables to evaluate any differences. When these steps did not alter the results, the final report included analyses using the non-transformed variables and all data points.

When the linear mixed model showed a significant main effect of time, post-hoc pairwise comparisons were calculated to examine differences between all time points (within-group). In case of a significant interaction term, the between-group differences at all time points were compared as well. A multivariate t correction of the p-values for multiple testing was applied in these post hoc pairwise comparisons. The effect sizes were reported using Cohen’s D values. The models were additionally run with covariates gender and work regime (working fulltime vs. less than fulltime prior to retirement) to examine whether the conclusions differ after controlling for these two factors.

Finally, sensitivity analyses with imputed data were performed using an iterative Markov chain Monte Carlo (MCMC) method [45]. The same analysis methods applied to the original data with missing values were then conducted on the imputed data and the results were compared.

A post hoc power analysis was performed using G*power (version 3.1) based on a repeated measures ANOVA design (within factors) with α = 0.05 and desired power (1-β) = 0.80 to determine the effect size that could be detected with the given sample size.

## Results

**Fig. 1:**
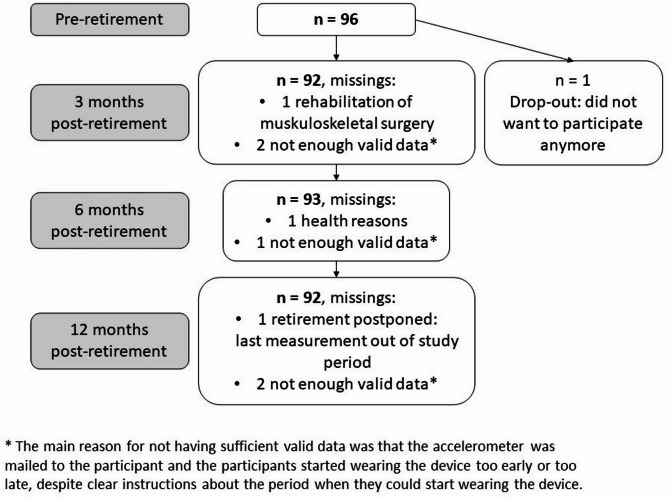
Participant flow

In total, 96 participants were measured pre-retirement (Fig. 1: participant flow). The characteristics of the participants are presented in Table 1, indicating that the majority were women (59%) and had higher SEP (59% with high education, 77% non-manual workers and 49% with higher post-retirement income).

**Table 1:** Sample characteristics

Pre-retirement (*n* = 96)		Median (IQR)
Age at retirement		62 (3.52)
Mc Arthur scale of subjective social status (0–10) [65]		7 (2)
		**N (%)**
Gender	Women	57 (59)
	Men	39 (41)
Level of education	ISCED^1^ 0–4: low-intermediate education	39 (41)
	ISCED 5–8: high education	57 (59)
Occupational group	ISCO^2^ 5–9: manual worker	22 (23)
	ISCO 0–4: non-manual worker	74 (77)
Living comfortably with income	With a lot of difficulty	0 (0)
	With difficulty	1 (1)
	Reasonably	10 (10)
	Good	48 (50)
	Very good	37 (39)
Partnership status	Life partner	81 (19)
Partner’s working status	(self-)employed	40 (42)
Children	No children	6 (6)
Grandchildren	No grandchildren	29 (30)
Percentage of fulltime employment	100	42 (44)
	80%	23 (24)
	50–80%	27 (28)
	< 50%	4 (4)
Subjective health	Very bad	0 (0)
	Bad	0 (0)
	Reasonable	11 (11)
	Good	73 (76)
	Very good	12 (13)
12 *months post-retirement* (n = 94)		
Equivalised disposable income	Below median	39 (41)
	Above median	46 (49)
	Missing answer	9 (10)
Reporting some paid work		18 (19)

^1^International standard classification of education

^2^International standard classification of occupations

### Changes in 24-h movement behaviors during the retirement transition: descriptive analysis

**Fig. 2:**
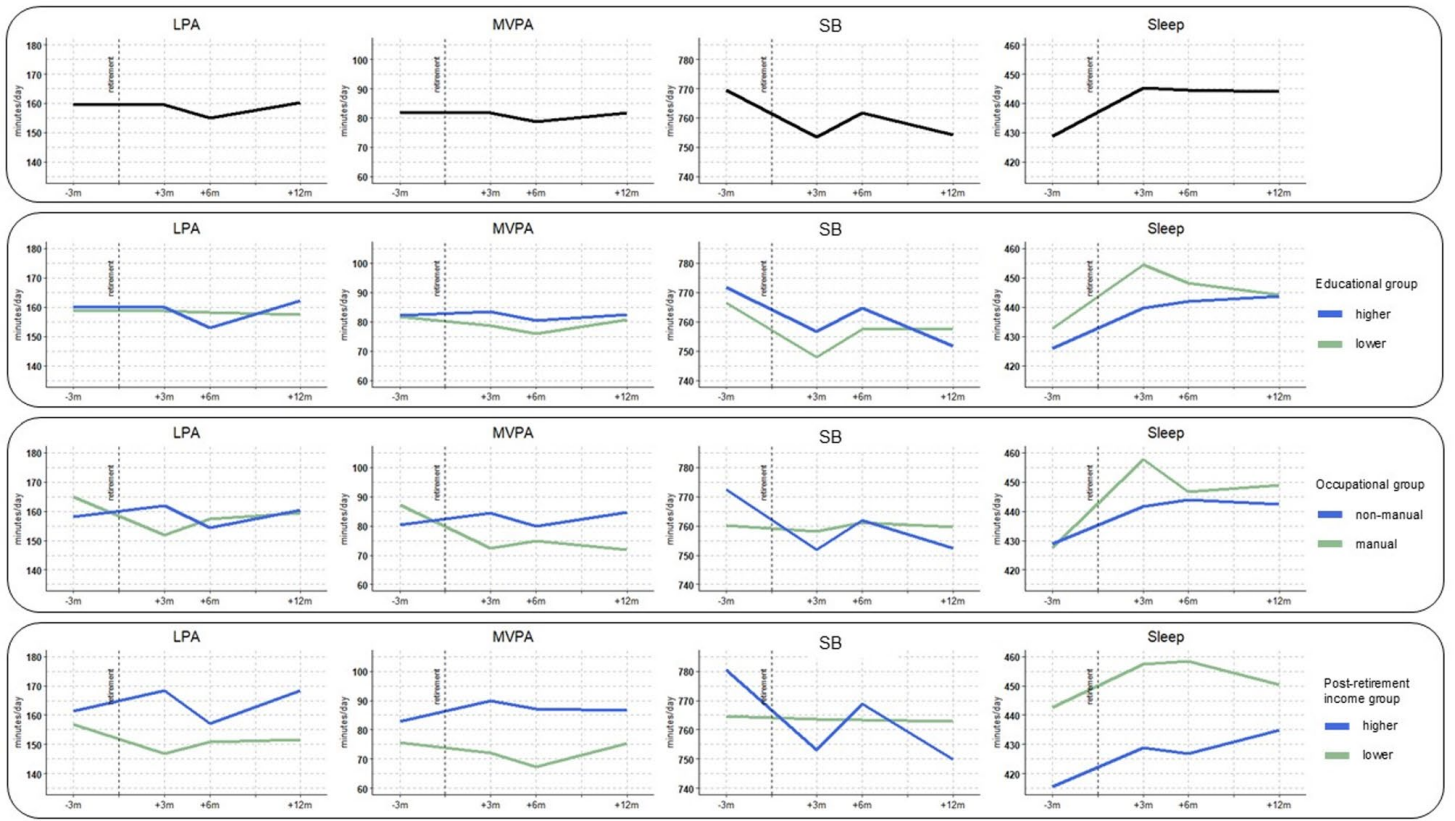
line plots for changes in the compositional means of the behaviours over time, for the whole sample and by SEP

The compositional means of the 24-h behaviors before and after retirement in minutes and the medians and interquartile ranges of the intensity gradient and average acceleration, as well as its changes, are represented in Supplementary file 4 and 5 and in Fig. 2 for the total sample and for the SEP groups separately. In the total sample, the composition pre-retirement included 74 min of MVPA, 157 min of LPA, 777 min (= 13.0 h) of SB and 433 min (= 7.2 h) of sleep on average per day. Among the whole study sample, the changes in MVPA and LPA were smallest (− 1.4.% and + 0% respectively), while the time spent inactive decreased from pre-retirement to 12 months after retirement by 16 min (− 2.1%) and the time spent in sleep increased by 15 min (+ 3.5%). As seen in Fig. 2, the most marked changes in sleep and SB occurred from pre- to three months post-retirement. These behaviors were relatively stable afterwards, especially sleep.

**Fig. 3:**
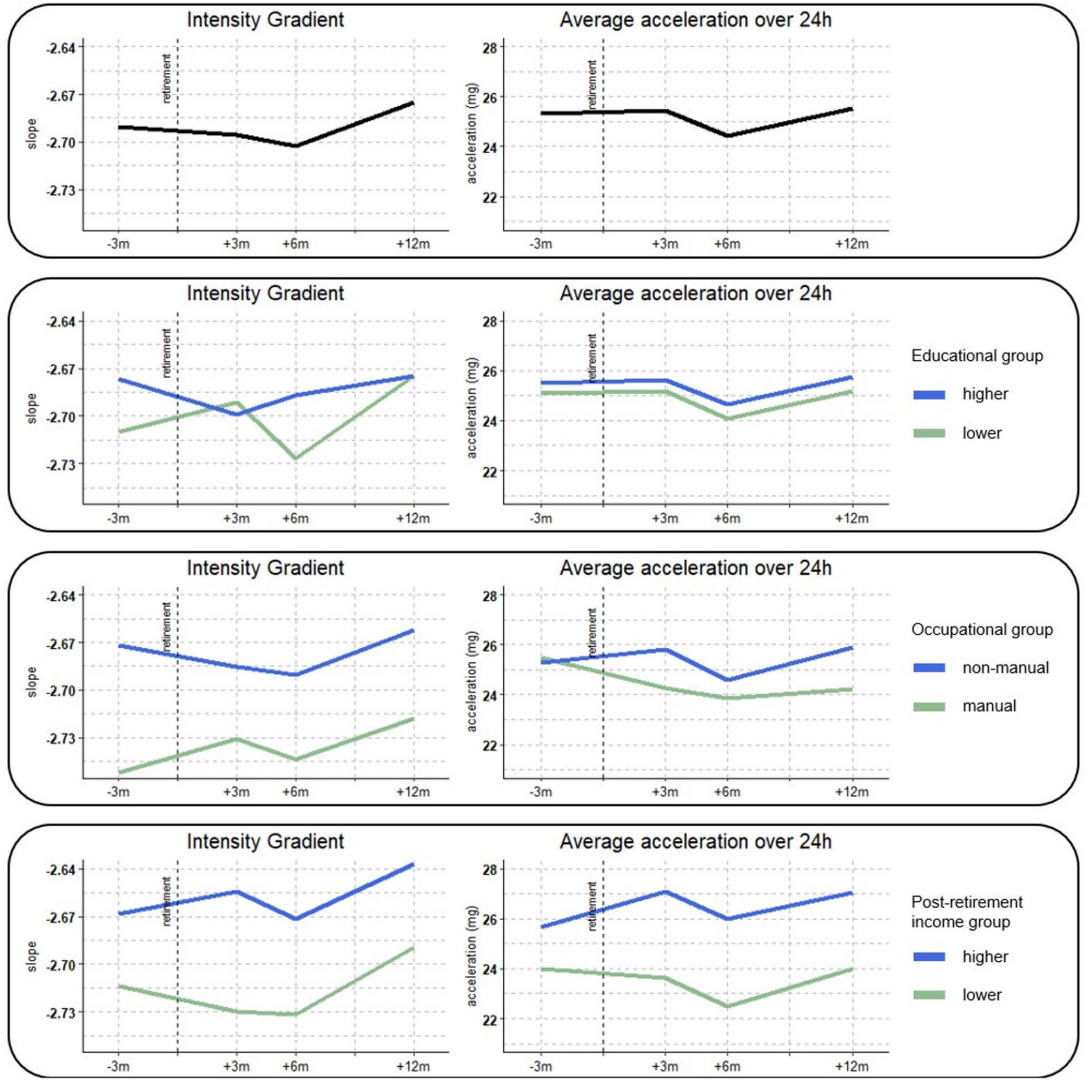
line plots for changes in the intensity gradient and average acceleration over time, for the whole sample and by SEP

The line plots for changes in the intensity gradient and average acceleration are presented in Fig. 3. The intensity gradient decreased slightly between pre- and six months post-retirement (indicating less intense PA), but increased from six to twelve months post-retirement (indicating more intense PA). The average acceleration was rather stable from pre- to three months post-retirement, decreased towards six months post-retirement and increased again to baseline levels by twelve months post-retirement.

### Changes in 24-h movement behaviors during the retirement transition: linear mixed models

The results of the linear mixed models can be found in Tables 2 and 3 and the detailed results of the post hoc tests are available in Supplementary file 6. In the models to predict changes in active versus passive behaviors (coordinate 1) and LPA vs. MVPA (coordinate 2), no statistically significant changes were found. However, there was a significant decrease in SB versus sleep (coordinate 3) from baseline to all the other time points and these differences were confirmed in the post hoc tests (pre-post3m: *p* = 0.005, ES = 0.49; pre-post6m: *p* = 0.047, ES = 0.38; pre-post12m: *p* = 0.011, ES = 0.46). This implies that there was a statistically significant increase in sleep duration relative to the decrease in SB. Finally, there were no statistically significant changes in the intensity gradient nor in average acceleration.

**Table 2:** Results of the linear mixed models to investigate the changes in the balance coordinates over time.

	Coordinate 1 Active (LPA, MVPA) vs. passive behaviors (SB, sleep)			Coordinate 2 LPA vs. MVPA			Coordinate 3 SB vs. sleep		
	β	95%CI	*R*²	β	95%CI	*R*²	β	95%CI	*R*²
*Time* Baseline vs.			0.002			0.000			0.013
3 m	− 0.03	[− 0.09;0.04]		0.01	[− 0.04;0.06]		− 0.04***	[− 0.07;− 0.02]	
6 m	− 0.04	[− 0.11;0.03]		− 0.01	[− 0.06;0.04]		− 0.03**	[− 0.06;− 0.01]	
12 m	0.00	[− 0.06;0.07]		− 0.01	[− 0.05;0.04]		− 0.04**	[− 0.06;− 0.01]	
Time*education Higher educ vs. lower educ for baseline vs.			0.003			0.004			0.016
3 m	0.03	[− 0.10;0.17]		− 0.05	[− 0.15;0.05]		0.01	[− 0.04;0.06]	
6 m	0.01	[− 0.13;0.15]		− 0.08	[− 0.17;0.02]		− 0.00	[− 0.05;0.05]	
12 m	0.03	[− 0.11;0.16]		0.00	[− 0.10;0.10]		− 0.02	[− 0.07;0.03]	
Time*occupation Non− manual vs. manual for baseline vs.			0.012			0.016			0.014
3 m	0.20*	[0.04;0.36]		− 0.07	[− 0.19;0.04]		0.01	[− 0.05;0.07]	
6 m	0.11	[− 0.05;0.27]		− 0.07	[− 0.19;0.04]		− 0.00	[− 0.06;0.06]	
12 m	0.18*	[0.017;0.34]		− 0.11	[− 0.22;0.01]		− 0.01	[− 0.07;0.05]	
Time*income Above median vs. below median for baseline vs.			0.054			0.038			0.034
3 m	0.16*	[0.02;0.30]		− 0.06	[− 0.16;0.04]		− 0.02	[− 0.07;0.03]	
6 m	0.10	[− 0.04;0.24]		− 0.10*	[− 0.20;− 0.00]		− 0.01	[− 0.06;0.05]	
12 m	0.08	[− 0.06;0.22]		− 0.01	[− 0.11;0.09]		− 0.05*	[− 0.10;− 0.00]	

The reported R^2^ is the marginal R^2^, which represents the explained variance of the fixed effects (the predictors) alone. *p < 0.05 **p < 0.01 ***p < 0.001.

**Table 3:** Results of the linear mixed models to investigate the changes in the intensity gradient and average acceleration over time.

	Intensity gradient			Average acceleration		
	β	95%CI	*R*²	β	95%CI	*R*²
*Time* Baseline vs.			0.003			0.004
3 m	− 0.01	[− 0.04;0.03]		0.09	[− 1.14;1.32]	
6 m	− 0.01	[− 0.05;0.03]		− 0.82	[− 2.05;0.40]	
12 m	0.02	[− 0.02;0.05]		0.24	[− 0.99;1.46]	
Time*occupation Non− manual vs. manual for baseline vs.			0.017			0.007
3 m	− 0.04	[− 0.13;0.04]		1.38	[− 1.54;4.30]	
6 m	− 0.03	[− 0.12;0.06]		0.73	[− 2.18;3.66]	
12 m	− 0.03	[− 0.12;0.06]		1.60	[− 1.32;4.5]	
Time*education Higher educ vs. Lower educ for baseline vs.			0.007			0.005
3 m	− 0.04	[− 0.11;0.04]		0.11	[− 2.40;2.63]	
6 m	0.01	[− 0.07;0.08]		0.05	[− 2.44;2.55]	
12 m	− 0.03	[− 0.11;0.04]		0.15	[− 2.35;2.65]	
Time*income Above median vs. below median for baseline vs.			0.030			0.053
3 m	0.03	[− 0.05;0.11]		1.64	[− 0.91;4.20]	
6 m	0.02	[− 0.06;0.09]		1.85	[− 0.69;4.39]	
12 m	0.01	[− 0.07;0.09]		1.44	[− 1.10;3.98]	

The reported R² is the marginal R^2^, which represents the explained variance of the fixed effects (the predictors) alone. *p < 0.05 **p < 0.01 ***p < 0.001.

### Changes in 24-h movement behaviors during the retirement transition by education: descriptive analysis

Based on the descriptive changes in compositional means, higher educated participants decreased SB more (− 21 min) and increased sleep slightly more (+ 17 min) compared to lower educated individuals (SB − 9 min; sleep + 11 min). The higher education group had a higher intensity gradient (i.e., more intense PA) at pre- and at six months post-retirement and a slightly higher average acceleration at all time points compared to the lower education group.

### Changes in 24-h movement behaviors during the retirement transition by education: linear mixed models

There were no statistically significant differences over time within lower nor higher educated groups in any of the coordinates, nor in the intensity gradient or average acceleration. There were also no significant differences between the education groups at any measurement time point.

### Changes in 24-h movement behaviors during the retirement transition by occupation: descriptive analysis

Non-manual workers seemed to slightly increase time spent in active behaviors (MVPA + 5 min; LPA + 3 min) and sleep (+ 13 min), with a decrease in SB (-21 min). In contrast, manual workers tended to decrease time spent in active behaviors (MVPA − 13 min; LPA − 9 min) and increase sleep more than non-manual workers (+ 20 min), while SB was stable (+ 1 min). Except for the pre-retirement average acceleration which was similar for both groups, the non-manual group had a higher intensity gradient and average acceleration at all time points.

### Changes in 24-h movement behaviors during the retirement transition by occupation: linear mixed models

The change in active vs. passive behaviors (coordinate 1) differed between non-manual and manual workers from baseline to post3m (*p* = 0.017) and from baseline to post12m (*p* = 0.030). However, in the post-hoc analyses, these within- and between-group differences were not statistically significant between any of the time points, implying that there are no substantial changes in any of the groups, and that the difference observed in the linear model is not relevant. Lastly, the changes in intensity gradient and average acceleration did not significantly differ by occupation.

### Changes in 24-h movement behaviors during the retirement transition by post-retirement income: descriptive analysis

Descriptively, there were differences between lower and higher income groups. From pre- to twelve months after retirement, the higher income group had a marked decrease in SB (-31 min) and increase in sleep (+ 20 min), whereas the lower income group had only modest changes in its movement behaviors (MVPA − 0 min; LPA − 5 min; SB -1 min; sleep + 6 min). The higher income group had a higher intensity gradient and average acceleration at all time points compared to the lower income group.

### Changes in 24-h movement behaviors during the retirement transition by post-retirement income: linear mixed models

The linear model revealed that the change in active vs. passive behaviors (coordinate 1) was different between lower and higher income groups from baseline to post3m (*p* = 0.026). At baseline, there was no difference in 24-h movement behaviors between the income groups. However, from baseline to post3m, the higher income group increased both MVPA (+ 7 min) and LPA (+ 6 min), and decreased SB (-28 min), while the low income group decreased both MVPA (-7 min) and LPA (-10 min) and did not change their SB (+ 1 min). These changes resulted in statistically significant differences in active vs. passive behaviors between the groups at post3m in post-hoc analyses (*p* = 0.016; ES=-1.10).

Additionally, the change in LPA vs. MVPA (coordinate 2) from baseline to post6m was significantly different between lower and higher income groups in the linear model (*p* = 0.043). The post hoc test also yielded a significant difference between the income groups at 6 months post-retirement (*p* = 0.027, ES = 0.98). Both income groups had similar decreases in LPA, while MVPA increased by 4 min in the higher income group and decreased by 7 min in the lower income group.

Finally, there was a significantly different change between income groups for SB vs. sleep (coordinate 3) from baseline to 12 months post-retirement (*p* = 0.046). However, the post hoc pairwise comparisons showed a significant change from baseline to 12 months post-retirement for the higher income group only (*p* = 0.005, ES = 0.75). The higher income group decreased SB by 31 min and increased sleep by 20 min, while the lower income group decreased SB only by 1 min and increased sleep only by 6 min daily. Lastly, the changes in intensity gradient and average acceleration did not significantly differ by post-retirement income.

### Additional analyses

First, all tests have been repeated as adjusted models with covariates gender and pre-retirement work regime (working fulltime vs. not working fulltime). This resulted in the same conclusions as the analysis without covariates. Secondly, the analyses were repeated with imputed data (Supplementary file 7).

## Discussion

The current longitudinal observational study investigated the role of SEP in the changes in 24-h movement behaviors during the retirement transition in Flemish adults over four time points using compositional data analysis (CoDA).

Concerning the changes in movement behaviors in the whole sample, not taking SEP into account, our hypothesis was partly confirmed. More specifically, sleep increased and SB decreased, in line with our hypothesis. By contrast, contrary to our hypothesis, PA did not decrease, but remained stable. The changes in sleep and SB happened mainly between pre-retirement and three months post-retirement, and were relatively stable afterwards, especially sleep.

When the role of SEP was taken into account, the SEP indicators education and occupation had no statistically significant association with the changes in movement behaviors in the present study, although descriptively, there was a trend in the higher SEP subgroups to change their lifestyle towards a more health-enhancing lifestyle. Nonetheless, when investigating post-retirement income as SEP indicator, there were several models showing significantly more favorable changes for the higher income group’s lifestyle, in line with our hypothesis. To start, the higher income group increased the proportion of active (LPA and MVPA) vs. passive behaviors (SB and sleep) from pre- to three months after retirement. More explicitly, the higher income group increased PA and decreased SB, while the lower income group decreased PA and did not change the time spent inactive, and sleep increased equally for both income groups. Secondly, the analyses showed a different change in LPA vs. MVPA between the income groups, since the higher income group increased MVPA, the lower income group decreased MVPA, while both groups decreased LPA similarly. This means that the higher income group shifted towards more intense PA, while the lower income group decreased the intensity of PA. Finally, the higher income group decreased the proportion of SB vs. sleep more than the lower income group. It needs to be mentioned that the R² values in our models were small: the highest was 0.054, which means that the predictor (SEP) explained only 5.4% of the variance in the outcome (the change in movement behaviors) (Table 2). This means that there are other factors that explain the remaining variance. This is not surprising, since movement behaviors as well as differences between SEP groups are parts of complex systems [46]. Also, the retirement transition is a life event that influences life in many ways, which adds to the complexity. Some of the other factors that might influence this model are gender, age, health, functional status, BMI. We propose to investigate these (further) in future studies.

We like to point out that the observed increase in sleep is similar to previous studies [5, 8, 9, 47]. Quantitative and qualitative evidence showed that retirees postpone their sleep onset and wake-up compared to pre-retirement, and that this postponed awakening explains the increase in sleep duration [8, 9, 48]. The sleep duration in the present study sample increased from 7 h to 13 min to 7 h and 28 min, which is still within the recommended range of 7–9 h [49]. Since we only investigated sleep duration, we do not know whether sleep quality changed. However, previous studies that investigated sleep disturbances and quality of sleep found that these sleep indicators improve as well during the retirement transition [50–52].

By contrast, the decrease in SB in the present study is somewhat different to other studies with device-based movement behavior measurements. Those studies reported smaller decrease in sedentary behavior [5, 7]. Suorsa et al. [53] even reported an increase of 24 min per day in SB, except in men from non-manual occupations, who had a (non-significant) decrease in sedentary behavior of 13 min per day. The different results in the present study might be explained by the fact that, compared to previous studies, the sample of the present study included proportionally more men with non-manual occupations. These men might have a stronger tendency to decrease their SB, although this should be investigated further. Findings from earlier studies based on self-reported sedentary behavior were consistent with the results of the current study, namely that total SB seemed to decrease. Studies investigating domain-specific SB concluded that this was due to a larger decrease in occupational SB than the increase in recreational sedentary activities [3].

When looking at the total sample, we observed little changes in PA. This finding is in contrast with previous studies investigating changes in device-based total PA, which did observe decreases in total PA [5–7]. However, the previous device-based studies were all conducted in Finland and Sweden, hence the context of the study might lead to different results [18]. Moreover, the previous studies included proportionally more women and more manual workers than the current study. Also, since all participants in the present study retired in 2022 or the beginning of 2023, Covid-19 lockdowns took place in their last working years and remote working was more common since then. This might have changed their PA patterns already prior to retirement, as some recent retirees explicitly mentioned in a recent qualitative study investigating the changes in barriers and facilitators for PA during the retirement transition [48]. Moreover, a systematic review based on 57 studies concluded a decrease in PA during the pandemic [54], which might not be fully compensated when the social restrictions decreased. Another study in Flemish adults suggests that the gap between sufficiently and insufficiently active individuals widened even more during the first lockdown [55]. Besides this, it might be that there were changes in PA that only occured after the follow-up period of this study, thus later than one year after retirement [2]. More investigation is needed to find out whether there might be a long adjustment period or ‘honeymoon period’ after retirement.

Finally, no changes in the intensity gradient or average acceleration were detected. This might be due to the fact that changes were mainly found in SB and sleep, which are both on the inactive side of the gradient and both lead to low acceleration signal. These results confirm that there were little changes in PA and PA intensity, independent of the use of cut-points between PA intensities, since the intensity gradient and average acceleration are not based on any cut-points.

In terms of SEP, we did identify differences between income groups in the changes in 24-h movement behaviors. In line with our hypothesis, they changed all more towards a health-enhancing lifestyle in the higher post-retirement income group versus in the lower income group. This means that there may be a need for strategies to improve the movement behaviors of lower SEP groups during this life transition, especially since they have worse health in general [56]. A decrease in financial resources and an increased awareness of the cost of PA when retiring has been mentioned as a barrier for PA in the qualitative study by McDonald et al. (2015) [57], but not in a qualitative study in Flanders [48]. It is interesting that not all SEP indicators seemed to play an equally important role. However, income is recognized as being more strongly associated with late-life health outcomes than education or occupation, as it reflects accumulated resources across the life course [58]. An even better indicator of SEP in late life could be wealth, but this is complex to measure and was therefore not included in the present study. Education might be a weaker indicator in older adults, since social mobility might have played an important role in boosting employment opportunities [59].

In this light, it is also important to mention that recent literature concerning occupational PA, which is more often performed by lower SEP adults, may not have the same favorable health implications compared to recreational PA (referred to as the PA paradox) [60, 61]. To the best of our knowledge, the health implications of replacing occupational PA by recreational PA when retiring have not been investigated yet.

The main strengths of the present study are the multiple SEP indicators, the device-based 24-h measures, the longitudinal design using four time points in a compositional data analysis, and the combination with an intensity cut-point free estimate of 24-h movement behaviors, namely the intensity gradient and average acceleration. Nevertheless, the present study is not without limitations. First of all, the sample is relatively small which limits the power of the analysis and generalisability of the results. A post hoc power calculation based on repeated measures ANOVA indicates that an effect size of 0.13 and larger could be detected. However, the interaction with SEP is not included in this power analysis. Also, manual workers are underrepresented, potentially due to recruitment and selection bias. These biases may also have resulted in a sample more interested in health and lifestyle than the target population.

Future studies should aim to recruit more participants and especially more lower SEP participants. A second limitation is the estimation of SB with a wrist-worn accelerometer. As this measurement provides no indication of the angle of the upper leg, it cannot truly identify sedentary behavior, which is by definition in a sitting, reclining or lying position [62]. Therefore, our measurement captured ‘inactivity’ rather than SB, but was called SB to be consistent with previous articles. However, the alternative location of a device to measure 24-h movement behaviors is a thigh-worn accelerometer, which is much more invasive and might hinder recruitment and retention of participants as well as wear time per day [25]. A third limitation refers to the applied cut-points to estimate time spent in the different movement behaviors. These cut-points are based on small validation studies with adults aged 21–61 years old. However, to the best of our knowledge, there are no cut-points available for the age group in the present study that are based on larger samples. Moreover, the other candidate cut-points are very similar to the currently used cut-points [63, 64]. However, without denying this latter limitation, we were mainly interested in changes in movement behaviors and not in absolute values, so the consistency of measurement may be more important than the validity of the absolute values. Moreover, we included the intensity gradient and average acceleration as cut-point free estimates of 24-h movement behaviors to complement the analysis based on the cut-point based estimates. As a final limitation, the variability in the timing of the pre-retirement measurement from one to six months before retirement also constitutes a limitation of the present study. When measuring participants at six months prior and six months after retirement, the seasonal effects are controlled at least between two time points. Unfortunately, due to practical reasons, this was not possible in the present study.

## Conclusion

In conclusion, the current study found that changes in 24-h movement behaviour during the retirement transition in general were favorable for health, since PA was stable, SB decreased and sleep time increased. However, the changes were less favorable for adults with lower SEP, especially those with a lower post-retirement income. Retirees with lower SEP might benefit from lifestyle interventions during the retirement transition, as these individuals are generally at a higher risk for health conditions compared to retirees from higher SEP populations.

## Supplementary Information

Below is the link to the electronic supplementary material.

Supplementary Material 1.

Supplementary Material 2.

Supplementary Material 3.

Supplementary Material 4.

Supplementary Material 5.

Supplementary Material 6.

Supplementary Material 7.

## Data Availability

The data used to support the findings of this study are available from the corresponding author upon reasonable request.
