# Supplementary material for: Socio-economic position and changes in 24-h movement behaviors during the retirement transition
Source: J Act Sedentary Sleep Behav. 2025 Oct 16;4:17. doi: 10.1186/s44167-025-00087-7 (PMC12532850; doi:10.1186/s44167-025-00087-7)
Supplement: Supplementary file 2 — Supplementary Material 2. [file 44167_2025_87_MOESM2_ESM.pdf]

median equivalised income Flanders 2023 (statbel)

€ 31,102.00 yearly

€ 2,591.83 monthly

this falls in the yellow categories

couples

answer options in our questionnaire (disposable household income)

N										
0	between	€ 0.00	and	€ 1,249.00	divided by 1.5 -->	between	€ 0.00	and	€ 832.67	lower income
0	between	€ 1,250.00	and	€ 1,499.00		between	€ 833.33	and	€ 999.33	lower income
2	between	€ 1,500.00	and	€ 1,999.00		between	€ 1,000.00	and	€ 1,332.67	lower income
6	between	€ 2,000.00	and	€ 2,499.00		between	€ 1,333.33	and	€ 1,666.00	lower income
5	between	€ 2,500.00	and	€ 2,999.00		between	€ 1,666.67	and	€ 1,999.33	lower income
15	between	€ 3,000.00	and	€ 3,499.00		between	€ 2,000.00	and	€ 2,332.67	lower income
28	between	€ 3,500.00	and	€ 4,499.00		between	€ 2,333.33	and	€ 2,999.33	higher income
13	between	€ 4,500.00	and	more		between	€ 3,000.00	and	more	higher income
**69**										

singles

answer options in our questionnaire (disposable household income)

N					
1	between	€ 0.00	and	€ 1,249.00	lower income
1	between	€ 1,250.00	and	€ 1,499.00	lower income
6	between	€ 1,500.00	and	€ 1,999.00	lower income
3	between	€ 1.00	and	€ 2,499.00	lower income
5	between	€ 2,500.00	and	€ 2,999.00	higher income
0	between	€ 3,000.00	and	€ 3,499.00	higher income
0	between	€ 3,500.00	and	€ 4,499.00	higher income
0	between	€ 4,500.00	and	more	higher income
**16**					

Did not answer this question: n = 9 (mainly higher educated, genderbalanced, with partner)

dropout/missings at 12m post-retirement: n = 2

final sample who answered this question: n = 69+16=**85**
