# Supplementary material for: Socio-economic position and changes in 24-h movement behaviors during the retirement transition
Source: J Act Sedentary Sleep Behav. 2025 Oct 16;4:17. doi: 10.1186/s44167-025-00087-7 (PMC12532850; doi:10.1186/s44167-025-00087-7)
Supplement: Supplementary file 3 — Supplementary Material 3. [file 44167_2025_87_MOESM3_ESM.docx]

The raw data were analysed with the R package GGIR, version 3.0.5, which includes validated methods to analyze multiday accelerometer data and provides estimates of PA, inactivity and sleep parameters (1-4). Auto-calibration was performed and it was checked whether all calibration errors were smaller than 0.02 after calibration. Data from the first midnight until the last midnight were used, resulting in in a maximum of seven full 24-hour windows. The non-wear detection approach ‘2013’ was used (5, 6). This algorithm classifies non-wear time using 15-minute time blocks, based on the characteristics of the 60 min time window centered around these 15 minutes. More specifically, a 15-minute time block was classified as non-wear time if 1) if the value range, for at least two out of three axes, was less than 50 m*g* and 2) in the 60 min window around it, the standard deviation was less than 3.0 m*g* (milligravity; 1 m*g*=0.00981 m/s²) for at least two out of the three axes. Non-wear time was imputed based on the other measurement days, which is default in GGIR. A sleep diary provided by the participants was used in combination with the algorithm. Sleep time was defined as periods between the ‘lights out’ time and ‘wake up’ time in the sleep log, in which the change in the angle of the arm relative to the horizontal plane was less than 5 degrees for at least 5 minutes (3). When no sleep log was available for a night, the HDCZA algorithm of GGIR detected the sleep periods (4). Naps were not taken into account, since GGIR does not support this at the moment. Each measurement must include at least four valid days (24 hours) with 66% valid waking hours (default in GGIR), otherwise the measurement was excluded from the analysis.

1. Migueles JH, Rowlands AV, Huber F, Sabia S, van Hees VT. GGIR: A Research Community–Driven Open Source R Package for Generating Physical Activity and Sleep Outcomes From Multi-Day Raw Accelerometer Data. Journal for the Measurement of Physical Behaviour. 2019;2(3):188-96.

2. van Hees VT, Fang Z, Langford J, Assah F, Mohammad A, da Silva IC, et al. Autocalibration of accelerometer data for free-living physical activity assessment using local gravity and temperature: an evaluation on four continents. J Appl Physiol (1985). 2014;117(7):738-44.

3. van Hees VT, Sabia S, Anderson KN, Denton SJ, Oliver J, Catt M, et al. A Novel, Open Access Method to Assess Sleep Duration Using a Wrist-Worn Accelerometer. PLoS One. 2015;10(11):e0142533.

4. van Hees VT, Sabia S, Jones SE, Wood AR, Anderson KN, Kivimäki M, et al. Estimating sleep parameters using an accelerometer without sleep diary. Sci Rep. 2018;8(1):12975.

5. van Hees VT, Gorzelniak L, Dean Leon EC, Eder M, Pias M, Taherian S, et al. Separating movement and gravity components in an acceleration signal and implications for the assessment of human daily physical activity. PLoS One. 2013;8(4):e61691.

6. van Hees VT, Renstrom F, Wright A, Gradmark A, Catt M, Chen KY, et al. Estimation of daily energy expenditure in pregnant and non-pregnant women using a wrist-worn tri-axial accelerometer. PLoS One. 2011;6(7):e22922.
