# Supplementary material for: Socio-economic position and changes in 24-h movement behaviors during the retirement transition
Source: J Act Sedentary Sleep Behav. 2025 Oct 16;4:17. doi: 10.1186/s44167-025-00087-7 (PMC12532850; doi:10.1186/s44167-025-00087-7)
Supplement: Supplementary file 4 — Supplementary Material 4. [file 44167_2025_87_MOESM4_ESM.docx]

|  | **Intensity gradient** | | **Average acceleration (m*g*)** | |
| --- | --- | --- | --- | --- |
| **Total sample** | **Median** | **IQR** | **Median** | **IQR** |
| Pre | -2.72 | 0.22 | 24.97 | 9.63 |
| Change | -0.01 | -0.02 | -0.12 | -1.15 |
| Percentage change | -0.3% | -10.4% | -0.5% | -12.0% |
| 3 months after retirement | -2.71 | 0.20 | 24.85 | 8.48 |
| Change | -0.01 | +0.04 | -1.58 | -0.29 |
| Percentage change | -0.3% | +21.7% | -6.4% | -3.4% |
| 6 months after retirement | -2.70 | 0.24 | 23.26 | 8.19 |
| Change | +0.01 | -0.03 | +1.43 | -0.44 |
| Percentage change | +0.2% | -11.6% | +6.1% | -5.3% |
| 12 months after retirement | -2.71 | 0.21 | 24.69 | 7.75 |
| Total change pre to 12 months after retirement | -0.01 | -0.01 | -0.27 | -1.88 |
| Percentage change | -0.4% | -3.8% | -1.1% | -24.2% |
| **Non-manual** | **Median** | **IQR** | **Median** | **IQR** |
| Pre | -2.70 | 0.25 | 24.97 | 9.79 |
| Change | -0.01 | -0.04 | +0.41 | -1.03 |
| Percentage change | -0.4% | -15.7% | +1.6% | -10.5% |
| 3 months after retirement | -2.69 | 0.21 | 25.38 | 8.76 |
| Change | +0.00 | +0.02 | -2.18 | -0.87 |
| Percentage change | +0.1% | +10.0% | -8.6% | -9.9% |
| 6 months after retirement | -2.69 | 0.23 | 23.20 | 7.89 |
| Change | –0.00 | -0.03 | +1.77 | -0.12 |
| Percentage change | -0.1% | -12.2% | +7.6% | -1.5% |
| 12 months after retirement | -2.69 | 0.20 | 24.96 | 7.77 |
| Total change pre to 12 months after retirement | –0.01 | -0.05 | -0.01 | -2.02 |
| Percentage change | -0.4% | -22.8% | -0.0% | -26.0% |
| **Manual** | **Median** | **IQR** | **Median** | **IQR** |
| Pre | -2.73 | 0.12 | 25.08 | 9.50 |
| Change | +0.02 | +0.05 | -1.50 | -3.58 |
| Percentage change | +0.8% | +41.7% | -6.0% | -37.7% |
| 3 months after retirement | -2.75 | 0.16 | 23.58 | 5.92 |
| Change | +0.01 | +0.07 | -0.10 | +5.12 |
| Percentage change | +0.5% | +44.2% | -0.4% | +86.5% |
| 6 months after retirement | -2.77 | 0.24 | 23.49 | 11.04 |
| Change | -0.03 | -0.05 | +0.09 | -2.23 |
| Percentage change | -1.2% | -21.7% | +0.4% | -20.2% |
| 12 months after retirement | -2.74 | 0.18 | 23.57 | 8.81 |
| Total change pre to 12 months after retirement | +0.00 | +0.07 | -1.51 | -0.69 |
| Percentage change | +0.1% | +37.5% | -6.4% | -7.8% |
| **Higher education** | **Median** | **IQR** | **Median** | **IQR** |
| Pre | -2.70 | 0.25 | 24.98 | 10.13 |
| Change | +0.01 | -0.03 | +0.51 | -1.57 |
| Percentage change | +0.3% | -12.3% | +2.1% | -15.5% |
| 3 months after retirement | -2.71 | 0.22 | 25.49 | 8.57 |
| Change | -0.01 | -0.02 | -1.65 | +0.03 |
| Percentage change | -0.5% | -7.2% | -6.5% | +0.3% |
| 6 months after retirement | -2.70 | 0.21 | 23.84 | 8.59 |
| Change | -0.00 | -0.03 | +1.18 | -0.80 |
| Percentage change | -0.1% | -15.0% | +5.0% | -9.3% |
| 12 months after retirement | -2.70 | 0.18 | 25.02 | 7.79 |
| Total change pre to 12 months after retirement | -0.01 | -0.08 | +0.05 | -2.34 |
| Percentage change | -0.2% | -44.6% | +0.2% | -30.0% |
| **Lower education** | **Median** | **IQR** | **Median** | **IQR** |
| Pre | -2.72 | 0.21 | 24.38 | 7.72 |
| Change | -0.01 | -0.06 | -0.21 | +0.94 |
| Percentage change | -0.5% | -26.5% | -0.9% | +12.2% |
| 3 months after retirement | -2.71 | 0.16 | 24.17 | 8.66 |
| Change | +0.00 | +0.09 | -1.54 | -0.13 |
| Percentage change | +0.1% | +60.0% | -6.4% | -1.5% |
| 6 months after retirement | -2.71 | 0.25 | 22.63 | 8.53 |
| Change | +0.01 | +0.00 | +1.56 | -0.01 |
| Percentage change | +0.5% | +0.4% | +6.9% | -0.1% |
| 12 months after retirement | -2.72 | 0.25 | 24.18 | 8.52 |
| Total change pre to 12 months after retirement | +0.00 | +0.04 | -0.20 | +0.80 |
| Percentage change | +0.1% | +15.3% | -0.8% | +9.4% |
| **Higher income** | **Median** | **IQR** | **Median** | **IQR** |
| Pre | -2.70 | 0.19 | 24.99 | 9.65 |
| Change | -0.04 | +0.07 | +1.13 | -0.73 |
| Percentage change | -1.4% | +35.3% | +4.5% | -7.6% |
| 3 months after retirement | -2.66 | 0.25 | 26.11 | 8.92 |
| Change | +0.01 | +0.03 | -0.90 | +2.09 |
| Percentage change | +0.5% | +12.6% | -3.5% | +23.5% |
| 6 months after retirement | -2.67 | 0.29 | 25.21 | 11.01 |
| Change | +0.01 | -0.05 | +1.31 | -3.29 |
| Percentage change | +0.3% | -15.8% | +5.2% | -29.8% |
| 12 months after retirement | -2.68 | 0.24 | 26.51 | 7.73 |
| Total change pre to 12 months after retirement | -0.02 | +0.05 | +1.53 | -1.93 |
| Percentage change | -0.7% | +22.1% | +5.8% | -24.9% |
| **Lower income** | **Median** | **IQR** | **Median** | **IQR** |
| Pre | -2.73 | 0.25 | 23.80 | 8.38 |
| Change | +0.02 | -0.07 | -0.45 | -0.25 |
| Percentage change | +0.9% | -30.2% | -1.9% | -2.9% |
| 3 months after retirement | -2.75 | 0.17 | 23.35 | 8.14 |
| Change | -0.00 | +0.02 | -1.03 | -2.07 |
| Percentage change | -0.2% | +10.5% | -4.4% | -25.4% |
| 6 months after retirement | -2.74 | 0.19 | 22.32 | 6.07 |
| Change | -0.03 | +0.01 | +1.17 | +1.58 |
| Percentage change | -1.0% | +3.7% | +5.2% | +26.0% |
| 12 months after retirement | -2.72 | 0.20 | 23.49 | 7.64 |
| Total change pre to 12 months after retirement | -0.01 | -0.05 | -0.31 | -0.74 |
| Percentage change | -0.3% | -25.0% | -1.3% | -9.7% |
