# Supplementary material for: Socio-economic position and changes in 24-h movement behaviors during the retirement transition
Source: J Act Sedentary Sleep Behav. 2025 Oct 16;4:17. doi: 10.1186/s44167-025-00087-7 (PMC12532850; doi:10.1186/s44167-025-00087-7)
Supplement: Supplementary file 5 — Supplementary Material 5. [file 44167_2025_87_MOESM5_ESM.docx]

| **Total sample** | **MVPA** | **LPA** | **SB** | **Sleep** |
| --- | --- | --- | --- | --- |
| Pre | 74 | 157 | 777 | 433 |
| Change | -1 (-1.4%) | -1 (-0.6%) | -15 (-1.9%) | +18 (+4.2%) |
| 3 months after retirement | 72 | 156 | 761 | 451 |
| Change | -1 (-1.4%) | -5 (-3.2%) | +8 (+1.1%) | -2 (-0.4%) |
| 6 months after retirement | 71 | 151 | 769 | 448 |
| Change | +3 (+4.2%) | +6 (+4.0%) | -9 (-1.2%) | -1 (-0.2%) |
| 12 months after retirement | 75 | 157 | 761 | 448 |
| Total change pre to 12 months after retirement | +1 (-1.4%) | +0 (+0%) | -16 (-2.1%) | +15 (+3.5%) |
| **Non-manual** | **MVPA** | **LPA** | **SB** | **Sleep** |
| Pre | 73 | 155 | 779 | 433 |
| Change | +3 (+4.1%) | +3 (+1.9%) | -20 (-2.6%) | +14 (+3.2%) |
| 3 months after retirement | 76 | 158 | 760 | 447 |
| Change | -2 (-2.6%) | -8 (-5.1%) | +10 (+1.3%) | +0 (+0%) |
| 6 months after retirement | 73 | 150 | 769 | 447 |
| Change | +5 (+6.8%) | +7 (+4.7%) | -11 (-1.4%) | -1 (-0.2%) |
| 12 months after retirement | 79 | 157 | 758 | 446 |
| Total change pre to 12 months after retirement | +5 (+6.8%) | +3 (+1.9%) | -21 (-2.7%) | +13 (+3.0%) |
| **Manual** | **MVPA** | **LPA** | **SB** | **Sleep** |
| Pre | 76 | 164 | 768 | 432 |
| Change | -15 (-19.7%) | -15 (-9.1%) | -1 (-0.1%) | +30 (+6.9%) |
| 3 months after retirement | 61 | 149 | 767 | 463 |
| Change | +3 (+4.9%) | +4 (+2.7%) | +3 (+0.4%) | -10 (-2.2%) |
| 6 months after retirement | 64 | 153 | 770 | 453 |
| Change | -2 (-3.1%) | +2 (+1.3%) | -1 (-0.1%) | +0 (+0%) |
| 12 months after retirement | 63 | 155 | 769 | 453 |
| Total change pre to 12 months after retirement | -13 (-17.1%) | -9 (-5.5%) | +1 (+0.1%) | +20 (+4.6%) |
| **Higher education** | **MVPA** | **LPA** | **SB** | **Sleep** |
| Pre | 73 | 157 | 779 | 430 |
| Change | +0 (+0%) | -1 (-0.6%) | -14 (-1.8%) | +15 (+3.5%) |
| 3 months after retirement | 73 | 156 | 765 | 446 |
| Change | +0 (+0%) | -7 (-4.5%) | +7 (+0.9%) | -0 (-0%) |
| 6 months after retirement | 73 | 149 | 772 | 446 |
| Change | +2 (+2.7%) | +10 (+6.7%) | -14 (-1.8%) | +2 (+0.4%) |
| 12 months after retirement | 75 | 159 | 759 | 448 |
| Total change pre to 12 months after retirement | +2 (+2.7%) | +2 (+1.3%) | -21 (-2.7%) | +17 (+4.0%) |
| **Lower education** | **MVPA** | **LPA** | **SB** | **Sleep** |
| Pre | 74 | 156 | 773 | 436 |
| Change | -4 (-5.4%) | -0 (-0%) | -18 (-2.3%) | +22 (+5.0%) |
| 3 months after retirement | 70 | 156 | 755 | 459 |
| Change | -2 (-2.9%) | -2 (-1.3%) | +10 (+1.3%) | -6 (-1.3%) |
| 6 months after retirement | 68 | 154 | 765 | 453 |
| Change | +6 (+8.8%) | +0 (+0%) | -1 (-0.1%) | -5 (-1.1%) |
| 12 months after retirement | 74 | 155 | 764 | 447 |
| Total change pre to 12 months after retirement | -1 (-1.4%) | -2 (-1.3%) | -9 (-1.2%) | +11 (+2.5%) |
| **Higher income** | **MVPA** | **LPA** | **SB** | **Sleep** |
| Pre | 76 | 158 | 787 | 419 |
| Change | +7 (+9.2%) | +6 (+3.8%) | -28 (-3.6%) | +14 (+3.3%) |
| 3 months after retirement | 83 | 165 | 759 | 433 |
| Change | -3 (-3.6%) | -12 (-7.3%) | +17 (+2.2%) | -3 (-0.7%) |
| 6 months after retirement | 80 | 153 | 778 | 431 |
| Change | +2 (+2.5%) | +11 (+7.2%) | -21 (-2.7%) | +8 (+1.9%) |
| 12 months after retirement | 82 | 164 | 755 | 438 |
| Total change pre to 12 months after retirement | +6 (+7.9%) | +6 (+3.8%) | -31 (-3.9%) | +20 (+4.8%) |
| **Lower income** | **MVPA** | **LPA** | **SB** | **Sleep** |
| Pre | 68 | 154 | 771 | 447 |
| Change | -7 (-10.3%) | -10 (-6.5%) | +1 (+0.1%) | +16 (+3.6%) |
| 3 months after retirement | 61 | 144 | 773 | 463 |
| Change | -0 (-0%) | +3 (+2.1%) | -2 (-0.3%) | -0 (-0%) |
| 6 months after retirement | 61 | 146 | 770 | 462 |
| Change | +7 (+11.5%) | +3 (+2.1%) | -0 (-0%) | -9 (-1.9%) |
| 12 months after retirement | 68 | 149 | 770 | 453 |
| Total change pre to 12 months after retirement | -0 (-0%) | -5 (-3.2%) | -1 (-0.1%) | +6 (+1.3%) |

Table: compositional means of the 24-hour behaviours before and after retirement in minutes. The means are rescaled to a total of 1440 minutes and rounded to one minute.
