# Supplementary material for: Socio-economic position and changes in 24-h movement behaviors during the retirement transition
Source: J Act Sedentary Sleep Behav. 2025 Oct 16;4:17. doi: 10.1186/s44167-025-00087-7 (PMC12532850; doi:10.1186/s44167-025-00087-7)
Supplement: Supplementary file 6 — Supplementary Material 6. [file 44167_2025_87_MOESM6_ESM.docx]

| **Coordinate 3 (SB vs. sleep) – change over time** | | | |
| --- | --- | --- | --- |
|  | **Difference** | **P** | **ES** |
| Pre-post3m | 0.04 | 0.0053** | 0.4916 |
| Pre-post6m | 0.03 | 0.0470* | 0.3830 |
| Pre-post12m | 0.04 | 0.0105* | 0.4604 |
| Post3m-post6m | -0.01 | 0.8842 | -0.1085 |
| Post3m-post12m | -0.00 | 0.9968 | -0.0311 |
| Post6m-post12m | 0.01 | 0.9537 | 0.0774 |

|  | **Coordinate 1 (active vs. passive behaviors) – change over time including interaction term time*occupation** | | |
| --- | --- | --- | --- |
|  | **Difference** | **P** | **ES** |
|  | Manual workers | | |
| Pre-post3m | 0.18 | 0.1382 | 0.7589 |
| Pre-post6m | 0.13 | 0.4939 | 0.5507 |
| Pre-post12m | 0.13 | 0.4542 | 0.5686 |
| Post3m-post6m | -0.05 | 0.9917 | -0.2083 |
| Post3m-post12m | -0.04 | 0.9949 | -0.1903 |
| Post6m-post12m | 0.00 | 1.0000 | 0.0179 |
|  | Non-manual workers | | |
| Pre-post3m | -0.02 | 0.9986 | -0.0812 |
| Pre-post6m | 0.02 | 0.9990 | 0.0758 |
| Pre-post12m | -0.04 | 0.8838 | -0.1934 |
| Post3m-post6m | 0.04 | 0.9561 | 0.1570 |
| Post3m-post12m | -0.03 | 0.9923 | -0.1122 |
| Post6m-post12m | -0.06 | 0.6242 | -0.2693 |
|  | **Difference** | **P** | **ES** |
| Pre | 0.0560 | 0.9262 | 0.2411 |
| Post3m | -0.1392 | 0.4080 | -0.5991 |
| Post6m | -0.0543 | 0.9353 | -0.2338 |
| Post12m | -0.1210 | 0.5212 | -0.5210 |

| **Coordinate 1 (active vs. passive behaviors) – change over time**  **including interaction time*income** | | | |
| --- | --- | --- | --- |
|  | **Difference** | **P** | **ES** |
|  | Below the median income | | |
| Pre-post3m | 0.10 | 0.3529 | 0.4593 |
| Pre-post6m | 0.10 | 0.4524 | 0.4199 |
| Pre-post12m | 0.02 | 0.9991 | 0.1039 |
| Post3m-post6m | -0.01 | 1.0000 | -0.0394 |
| Post3m-post12m | -0.08 | 0.6631 | -0.3554 |
| Post6m-post12m | -0.07 | 0.7702 | -0.3159 |
|  | Above the median income | | |
| Pre-post3m | -0.05 | 0.8991 | -0.2378 |
| Pre-post6m | -0.00 | 1.0000 | -0.0151 |
| Pre-post12m | -0.05 | 0.9080 | -0.2311 |
| Post3m-post6m | 0.05 | 0.9282 | 0.2227 |
| Post3m-post12m | 0.00 | 1.0000 | 0.0067 |
| Post6m-post12m | -0.05 | 0.9354 | -0.2160 |
|  | **Difference** | **P** | **ES** |
| Pre | -0.09 | 0.6060 | -0.4074 |
| Post3m | -0.25 | 0.0162* | -1.1045 |
| Post6m | -0.19 | 0.0887 | -0.8424 |
| Post12m | -0.17 | 0.1532 | -0.7424 |

| **Coordinate 2 (LPA vs. MVPA) – change over time**  **including interaction term time*income** | | | |
| --- | --- | --- | --- |
|  | **Difference** | **P** | **ES** |
|  | Below the median income | | |
| Pre-post3m | -0.03 | 0.9800 | -1.825 |
| Pre-post6m | -0.04 | 0.8676 | -0.2714 |
| Pre-post12m | 0.01 | 0.9996 | 0.0906 |
| Post3m-post6m | -0.01 | 0.9996 | -0.0889 |
| Post3m-post12m | 0.04 | 0.8711 | 0.2731 |
| Post6m-post12m | 0.06 | 0.6391 | 0.3620 |
|  | Above the median income | | |
| Pre-post3m | 0.03 | 0.9750 | 0.1766 |
| Pre-post6m | 0.06 | 0.5594 | 0.3598 |
| Pre-post12m | 0.02 | 0.9926 | 0.1388 |
| Post3m-post6m | 0.03 | 0.9717 | 0.1832 |
| Post3m-post12m | -0.01 | 1.0000 | -0.0378 |
| Post6m-post12m | -0.04 | 0.9283 | -0.2210 |
|  | **Difference** | **P** | **ES** |
| Pre | 0.06 | 0.6906 | 0.3526 |
| Post3m | 0.12 | 0.1558 | 0.7117 |
| Post6m | 0.16 | 0.0274* | 0.9837 |
| Post12m | 0.07 | 0.6011 | 0.4007 |

| **Coordinate 3 (SB vs. sleep) – change over time**  **including interaction term time*income** | | | |
| --- | --- | --- | --- |
|  | **Difference** | **P** | **ES** |
|  | Below the median income | | |
| Pre-post3m | 0.02 | 0.8590 | 0.2781 |
| Pre-post6m | 0.02 | 0.8167 | 0.2945 |
| Pre-post12m | 0.01 | 0.9964 | 0.1324 |
| Post3m-post6m | 0.00 | 1.0000 | 0.0164 |
| Post3m-post12m | -0.01 | 0.9940 | -0.1457 |
| Post6m-post12m | -0.01 | 0.9892 | -0.1621 |
|  | Above the median income | | |
| Pre-post3m | 0.05 | 0.0893 | 0.5613 |
| Pre-post6m | 0.03 | 0.5436 | 0.3646 |
| Pre-post12m | 0.06 | 0.0052** | 0.7544 |
| Post3m-post6m | -0.02 | 0.9598 | -0.1967 |
| Post3m-post12m | 0.02 | 0.9627 | 0.1931 |
| Post6m-post12m | 0.03 | 0.4656 | 0.3898 |
|  | **Difference** | **P** | **ES** |
| Pre | -0.06 | 0.1626 | -0.7226 |
| Post3m | -0.04 | 0.5499 | -0.4394 |
| Post6m | -0.05 | 0.2331 | -0.6525 |
| Post12m | -0.01 | 0.9949 | -1.006 |

Effect size reported is Cohen’s D effect size.

*p<0.05 **p<0.01 ***p<0.001
