# Supplementary material for: Socio-economic position and changes in 24-h movement behaviors during the retirement transition
Source: J Act Sedentary Sleep Behav. 2025 Oct 16;4:17. doi: 10.1186/s44167-025-00087-7 (PMC12532850; doi:10.1186/s44167-025-00087-7)
Supplement: Supplementary file 7 — Supplementary Material 7. [file 44167_2025_87_MOESM7_ESM.docx]

The analysis has been repeated with imputed values for missing data in the 24-hour behaviors. Eight post-retirement 24-hour behavior measurements were missing (Figure 1), while the income variable was missing for ten participants. These missings were completely at random based on the Little’s MCAR test (p > 0.05). After imputation and rerunning the linear mixed model analysis, the conclusions regarding the changes in (non-)movement behaviors by income groups changed. There was still a significant difference between income groups for active vs. passive behaviors (coordinate 1) from pre- to three months post-retirement. However, no significant difference between income groups for LPA vs. MVPA (coordinate 2) from pre- to six months post-retirement (p=0.114) was maintained. The effect size for this difference decreased from large (0.98) to medium (0.76). Similarly, no significant difference between income groups for SB vs. sleep from pre- to 12 months post-retirement (p=0.089) was maintained. However, the effect size for this difference was medium in the original analysis (0.76) and was similar after imputation (0.70).
